# IGF2BP3 overexpression predicts poor prognosis and correlates with immune infiltration in bladder cancer

**DOI:** 10.1186/s12885-022-10353-5

**Published:** 2023-02-03

**Authors:** Wei Huang, Lizhen Zhu, Haoxuan Huang, Yuanyuan Li, Gongxian Wang, Cheng Zhang

**Affiliations:** 1 Department of Urology, The First Affiliated Hospital of Nanchang University, Nanchang, 330000 Jiangxi China; 2 Department of Oncology, The First Affiliated Hospital of Nanchang University, Nanchang, 330000 Jiangxi China; 3 Department of Gastroenterology, The First Affiliated Hospital of Nanchang University, Nanchang, 330000 Jiangxi China

**Keywords:** IGF2BP3, Bladder cancer, PDL-1, Prognosis marker

## Abstract

**Background:**

IGF2BP3 expression is associated with poor prognosis in cancers of multiple tissue origins. However, the precise mechanism of its co-carcinogenic action in bladder cancer is unknown.

**Methods:**

We aimed to demonstrate the relationship between IGF2BP3 expression and pan-cancer using The Cancer Genome Atlas (TCGA) database. We next validated IGF2BP3 expression in the Gene Expression Omnibus (GEO) database (GSE3167). Receiver operating characteristic (ROC) curve analysis was used to evaluate the diagnostic values of IGF2BP3. Cox and logistic regression were used to explore the factors affecting the prognosis. Protein–protein interactions (PPIs) network was constructed by STRING. Enrichment analyses were performed to infer involved pathways and functional categories of IGF2BP3 using the cluster Profiler package. We applied single-sample gene set enrichment analysis (ssGSEA) algorithm and TIMER database to evaluate the expression level of immune genes.

**Results:**

Pan-cancer analyses reveal that IGF2BP3 was higher in most cancer types, including bladder cancer, and the same results were found in GSE3167. The area under the ROC curve of IGF2BP3 was 0.736, which indicated that IGF2BP3 may be a potential diagnostic biomarker. High IGF2BP3 expression was associated with poorer overall survival (OS) (*P* = 0.015). For validation, we collected 95 bladder cancer samples and found that IGF2BP3 expression was higher in bladder cancer tissues than that in non-tumor bladder tissues by immunohistochemistry staining. We found a positive correlation between the expression level of IGF2BP3 and the clinical stage of bladder cancer. Immunocyte infiltration analysis showed that high IGF2BP3 expression was correlated with regulating the infiltration level of immune cell, including neutrophil cells and macrophages. IGF2BP3 promotes migration and invasion of bladder cancer cells, while IGF2BP3 inhibition had the opposite effects. Higher IGF2BP3 expression was closely associated with advanced TNM stage.

**Conclusion:**

IGF2BP3 overexpression was related to disease progression and poor prognosis, as well as infiltration of immune cells in bladder cancer. IGF2BP3 can be a promising independent prognostic biomarker and potential treatment target for bladder cancer.

**Supplementary Information:**

The online version contains supplementary material available at 10.1186/s12885-022-10353-5.

## Introduction

Bladder cancer is one of the most common urological carcinomas worldwide. It is reported that the incidence of bladder cancer continues to increase [1]. Globally, bladder cancer incidence is the 9th and cancer-related deaths the 13th among all cancer [2]. Despite continuous advances in surgery and combined chemoradiotherapy and even immunotherapy, clinical outcomes remain poor [3]. In order to improve clinical outcomes and prognosis in bladder cancer patients, the identification of effective biomarkers for early detection and prognosis assessment for clinical therapy of bladder cancer is required.

Insulin-like growth factor II mRNA-binding protein 3 (IGF2BP3), also known as the IGF2BP3 protein, is predominantly localized in the cytoplasm [4]. IGF2BP3 functions as a post-transcriptional regulator that recruits target coding and non-coding transcripts to protein-RNA complexes and affect the expression and translation of target RNAs [5]. As a member of the IGF2BP-family, IGF2BP3 regulates a number of genes involved in cancer cell proliferation, invasion, metastasis, and tumorigenesis [6–10]. IGF2BP3 is also related to the stemness, metabolism, and immunity of cells [11, 12]. Many studies had shown that IGF2BP3 was overexpressed in various cancers, including gastric cancer, lymphoid malignancies, nasopharyngeal cancer, and renal cell carcinoma [13–16], and that its overexpression correlates with a poor prognosis for overall survival. The relationship between IGF2BP3 expression and chemotherapy resistance has received much attention in recent years, for example in ovarian cancer and colorectal cancer [17, 18]. Recent reports revealed that IGF2BP3 levels were associated with T classification and correlated with worse outcomes [19]. However, the role of IGF2BP3 in bladder cancer has not been well clarified.

**Fig. 1:**
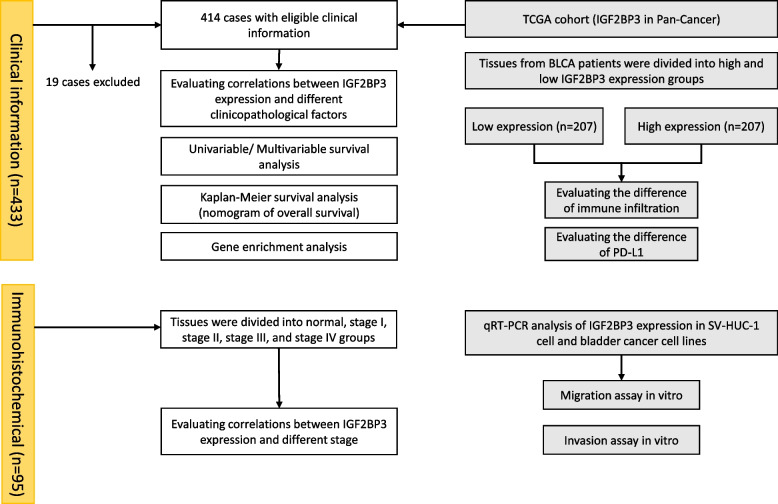
Workflow of this study

In this study, we analyzed gene expression data of IGF2BP3 from The Cancer Genome Atlas (TCGA), Gene Expression Omnibus (GEO), and Genotype-Tissue Expression databases to compare IGF2BP3 expressions in the tumor and normal tissues. Furthermore, we investigated the association of IGF2BP3 expression with clinicopathological features of bladder patients. We also addressed the relevance of IGF2BP3 expression to patient prognosis. In addition, gene function annotation, GO enrichment, KEGG enrichment, and Gene set enrichment analysis (GSEA) were carried out. Furthermore, we detected the relationship between IGF2BP3 expression and infiltrating immune cells. Finally, we detected IGF2BP3 expression in our bladder cancer samples and clarified the effect of IGF2BP3 on bladder cancer cell migration and invasion. Our study suggests that IGF2BP3 could be used as a prognosis marker in bladder cancer and promotes the progression through PD-L1. The workflow of our study is presented in Fig. 1.

## Materials and methods

### Data mining

By taking advantage of the Pan-Cancer project of the UCSC Xena (https://xenabrowser.net/datapages/), it is possible to comprehensively analyze the IGF2BP3 expression level in the pan-cancer. We analyzed the differential expression of IGF2BP3 in tumors and paired adjacent or normal tissues through TCGA databases, which combined the Genotype-Tissue Expression (GTEx) databases. GSE3167 (Platform: GPL96) was used to obtain bladder cancer microarray data from the GEO database.

### Correlation and gene set enrichment analysis

Differentially expressed genes (DEGs) were identified by the criteria of adjusted *P* value < 0.05 and log twofold change (FC) > 1.0. Gene expression correlation analysis was performed between IGF2BP3 and other mRNAs for given sets of TCGA expression data. The top 100 genes most positively correlated with IGF2BP3 were selected for enrichment analysis. To clarify the biological functions of IGF2BP3, GO functional and Kyoto Encyclopedia of Genes and Genomes (KEGG) pathway enrichment analyses were performed using the cluster profile package [20–22]. In order to identify biological pathways and functions associated with the changes in gene expression patterns, transcription profiles were analyzed by Gene Set Enrichment Analysis (GSEA).

### Survival prognosis analysis

Survival analyses of IGF2BP3 were conducted using the R packages ‘survival’ version 3.6.3. The median expression of IGF2BP3 within a cohort was used to stratify patients into low and high IGF2BP3 expression groups. For confirming the value of IGF2BP3 in predicting prognosis, we utilized the pROC of R packages (version 3.6.3) for receiver operating characteristic (ROC) statistics and ggplot2 (version 3.3.3) for generating plots.

### Immune cell infiltration analysis

To explore the specific association of IGF2BP3 with immune cells in bladder cancer, we used the single-sample GSEA computed with the GSVA package, and the deconvolution algorithm provided by the TIMER (tumor immune estimation resource) (http://timer.cistrome.org/) database for analysis. We inferred the effect of IGF2BP3 on tumor-infiltrating immune cell abundance from mRNA expression data using TIMER. Spearman’s rank correlation and p values based on the Wilcoxon test were calculated by statistical software R.

### Immunohistochemistry staining

In total, 95 patients who underwent surgery at the first affiliated hospital of Nanchang University from 2020 to 2021 for bladder cancer were included in the study. Written informed consent was obtained from each patient and the study was approved by the Ethics Committee of the first affiliated hospital of Nanchang University. The clinical features of bladder cancer patients included gender, age, TNM stage, histologic grade lymphovascular invasion and AJCC stage (Table S1). Formalin-fixed, paraffin-embedded samples were cut into 5 μm thick sections. Following washing, paraffin sections underwent antigen retrieval by heating the slides in a microwave in citrate or Tris/EDTA buffer. Endogenous peroxidase activity was blocked with 0.3% hydrogen peroxide in methanol. Tissue sections were blocked with blocking buffer (5% normal horse serum and 1% normal goat serum in PBS) and then incubated with an antibody against IGF2BP3 (1:100 dilution, Abcam, ab179807), CD3 (1:100 dilution, BOSTER, MA1015), CD68 (1:50 dilution, BOSTER, BM5785), CD16 (1:200 dilution, BOSTER, A01408-1), CD274 (1:50 dilution, BOSTER, BM4816) overnight at 4 °C, secondary antibody anti-rabbit (1:50 dilution, Beyotime, Shanghai, China) for 1 h at room temperature. The sections were stained with 3,3’-diaminobenzidine (DAB), then counterstained using hematoxylin. Images were taken using a Nikon Eclipse microscope (Nikon Americas Inc, NY).

### Cell culture

A normal human bladder epithelium cell line (SV-HUC-1) and bladder cancer cell lines (5637, J82, UMUC3 and T24) were purchased from the Cell Bank of Chinese Academy of Sciences (Shanghai, China). Cells were cultured in RPMI 1640 (Gibco, Carlsbad, CA) supplemented with 10% fetal bovine serum and penicillin/streptomycin with 5% CO2 at 37 °C.

### Lentivirus infection

The RNA interference sequence targeting the human IGF2BP3 gene (siRNA#1 GCAAAGGATTCGGAAACTT, siRNA#2 GGTGAAACTTGAAGCTCAT, siRNA#3 CCAGACACCTGATGAGAAT) or scramble siRNA as a negative control (NC) was inserted into the pLKO.1 lentiviral vectors. For overexpression assay, the coding sequence of IGF2BP3 mRNA was synthesized and cloned into the pLVX-Puro vector. The lentiviral vectors were transfected together with packing plasmids to 293 T cells by Lipofectamine 2000 (Invitrogen) and the supernatant was collected at 48 h after transfection. Gene expression was validated by qRT-PCR in lentivirus infected target cells.

### Quantitative real time PCR

Total RNA was isolated from cultured cells using TRIzol (Invitrogen). First strand cDNA was synthesized from total RNA using the Reverse Transcriptase Kit (Thermo, USA). SYBR Green PCR Master Mix (Thermo, USA) was utilized to perform quantitative real‐time PCR amplification according to the manufacturer's instructions. The primers for real‐time PCR are as follows: IGF2BP3 forward 5' GCACTTCCCTTTGTTGTAGTC 3', IGF2BP3 reverse 5' AGCACTTCCCTTAGGTTACTC 3'. Relative expression of IGF2BP3 was calculated as 2^−ΔΔCt^ using GAPDH as internal reference.

### Migration and invasion assays

Cell migration and invasion were assayed using a Transwell chamber (Corning, USA) with or without Matrigel. Briefly, cells (1.0 × 10^5^ cells per well) were seeded in the upper chamber of the 8.0μ pore size cell culture inserts that were either coated or uncoated with matrigel for migration and invasion assays respectively, and serum-containing media was placed into the lower chamber. After incubation for 24 h, non-migrating or invading cells on the surface of the upper chamber were removed with a cotton-tipped swab. The migrated and invaded cells on the lower surface of the membranes were fixed with 4% paraformaldehyde at room temperature for 30 min, stained with 0.1% crystal violet at room temperature for 30 min, and washed with PBS 3 times. The migrating or invading cell numbers were counted by a 200 × microscope.

### Statistics analysis

The bioinformatics analysis was conducted using R software (Version 3.6.3). All data are presented as mean ± standard deviation. Student’s t test was used to analyze the statistical significances of differences between two groups, and ANOVA to analyze the statistical significances of differences among multiple groups. The Fisher exact test, chi-square test, Wilcoxon signed-rank test, and logistic regression were used to estimate the correlation of IGF2BP3 expression and clinicopathologic features. The diagnostic accuracy was assessed by receiver operating characteristics (ROC) curve analysis. Kaplan–Meier method and Cox regression were used to evaluate the role of IGF2BP3 expression in prognosis. Statistical analyses for IHC and qRT-PCR were performed using GraphPad Prism v7.0d (GraphPad Software, Inc. San Diego, CA, USA) as indicated with *P* ≤ 0.05 considered to be statistically significant.

## Results

### Transcriptional levels of IGF2BP3 in pan-cancer

**Fig. 2:**
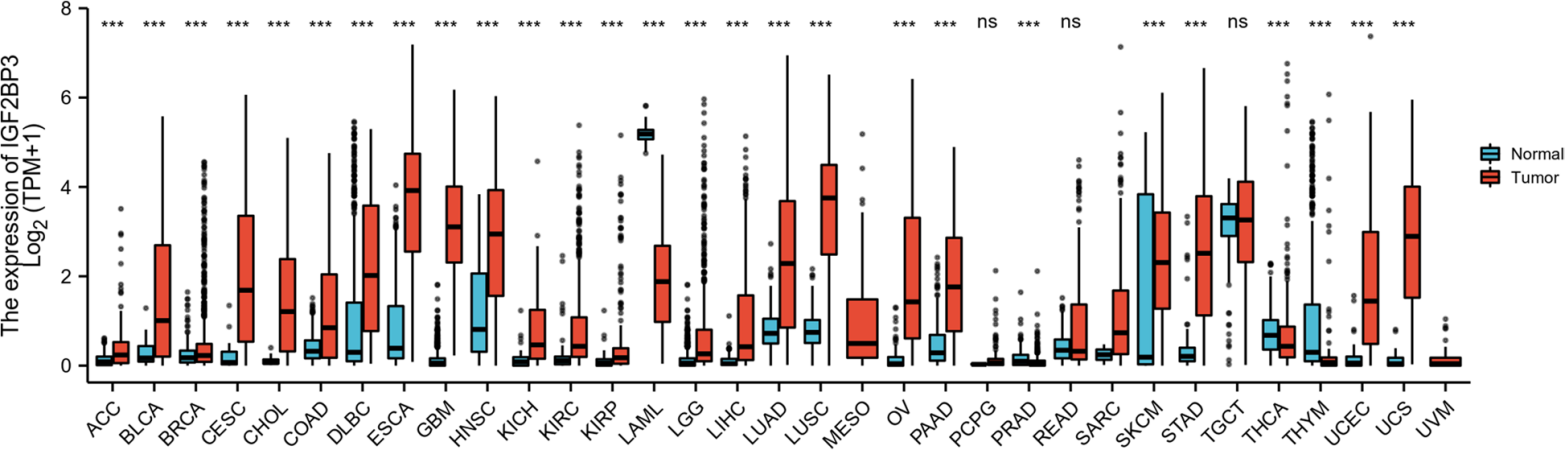
IGF2BP3 expression in normal and tumor tissues in TCGA and GTEx databases

**Fig. 3:**
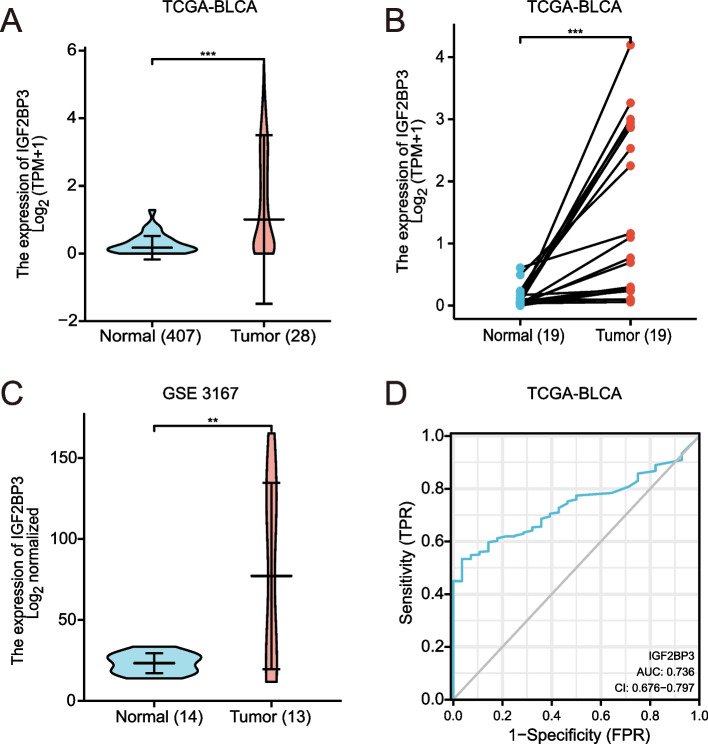
IGF2BP3 expression in TCGA and GEO database. **A** Expression of IGF2BP3 was frequently upregulated in 407 bladder tumor tissues compared with 28 normal bladder tissue samples in the TCGA profile. **B** The expression of IGF2BP3 in 19 paired bladder cancer samples from TCGA. **C** IGF2BP3 expression in 14 normal bladder tissues and 13 bladder tumor tissues from GSE3167. **D** ROC curve of IGF2BP3 in bladder cancer. X-axis represents false-positive rates, and Y-axis represents true-positive rates

IGF2BP3 is overexpressed by most human cancers, including ACC, BLCA, BRCA, CESC, CHOL, COAD, DLBC, ESCA, GBM, HNSC, KICH, KIRC, KIRP, LGG, LIHC, LUAD, LUSC, OV, PAAD, PRAD, SARC, SKCM, STAD, THCA, UCEC, and UCS. In bladder cancer, expression of IGF2BP3 was significantly upregulated in tumors compared to normal tissues, with a median level of 1.007 in tumor and 0.170 in normal tissue (*p* < 0.001) (Fig. 2). IGF2BP3 expression, however, was not significantly different between tumor and normal tissue in PCPG, READ, and TGCT. Besides, IGF2BP3 expression was low in LAML and THYM. IGF2BP3 mRNA expression was significantly upregulated in bladder tumor tissues compared to normal tissues from the TCGA-BLCA data sets (Fig. 3A-B). Next, we analyzed IGF2BP3 expression in one bladder cancer dataset (GSE3167, Platform: GPL96) retrieved from the GEO web database. This result is consistent with the data presented in TCGA, showing higher levels of IGF2BP3 expression in bladder cancer tissues than in noncancerous tissues (Fig. 3C). We then assessed the accuracy of IGF2BP3 predictions using the receiver operating characteristic (ROC) curve and the area under the ROC curve (AUC). We found that the area under the ROC curve (AUC) was 0.736 (Fig. 3D).

### Relationship between IGF2BP3 expression and clinical features of bladder cancer

Gene expression profiles and clinical data of 414 patients with bladder cancer were extracted from TCGA. Patients were divided into a high expression group (*n* = 207) and a low expression group (*n* = 207) using the median level (IGF2BP3) as the cutoff value. We next explored whether IGF2BP3 expression level was significantly associated with other clinicopathologic parameters. The results were shown in Table 1. There was a significant correlation between IGF2BP3 level with gender (*p* = 0.045). The overexpression of IGF2BP3 was positively correlated with late T stage (*p* = 0.003) and histologic grade (*p* < 0.001), but not with other parameters such as age, N stage, M stage, and lymphovascular invasion. Univariate logistic regression analysis was performed to further assess the association between IGF2BP3 expression and clinicopathological features of bladder cancer. On univariate logistic regression analysis, the IGF2BP3 expression level was significantly associated with gender (*p* = 0.*035) and T stage (p* = *0.002) *(Table 2)*.*

**Table 1:** Correlation analyzed between IGF2BP3 expression and clinicopathologic characteristics in bladder cancer based on TCGA database

Characteristic	Low expression of IGF2BP3	High expression of IGF2BP3	p
n	207	207	
Gender, n (%)			0.045
Female	45 (10.9%)	64 (15.5%)	
Male	162 (39.1%)	143 (34.5%)	
Age, n (%)			0.372
< = 70	122 (29.5%)	112 (27.1%)	
> 70	85 (20.5%)	95 (22.9%)	
T stage, n (%)			0.003
T1	5 (1.3%)	0 (0%)	
T2	73 (19.2%)	46 (12.1%)	
T3	87 (22.9%)	109 (28.7%)	
T4	30 (7.9%)	30 (7.9%)	
N stage, n (%)			0.407
N0	115 (31.1%)	124 (33.5%)	
N1	25 (6.8%)	21 (5.7%)	
N2	41 (11.1%)	36 (9.7%)	
N3	2 (0.5%)	6 (1.6%)	
M stage, n (%)			0.535
M0	117 (54.9%)	85 (39.9%)	
M1	5 (2.3%)	6 (2.8%)	
Histologic grade, n (%)			< 0.001
High Grade	185 (45%)	205 (49.9%)	
Low Grade	21 (5.1%)	0 (0%)	
Lymphovascular invasion, n (%)			1.000
No	63 (22.3%)	67 (23.7%)	
Yes	74 (26.1%)	79 (27.9%)

**Table 2:** IGF2BP3 expression associated with clinicopathologic characteristics by logistic regression

Characteristics	Total(N)	Odds Ratio(OR)	*P* value
Gender (Male vs. Female)	414	0.621 (0.397–0.964)	0.035
Age (> 70 vs. < = 70)	414	1.217 (0.825–1.798)	0.322
T stage (T3&T4 vs. T1&T2)	380	2.014 (1.303–3.142)	0.002
N stage (N2&N3 vs. N0&N1)	370	0.943 (0.580–1.532)	0.813
M stage (M1 vs. M0)	213	1.652 (0.482–5.900)	0.420
Histologic grade (High Grade vs. Low Grade)	411	0.97 (0.649–1.494)	0.988
Lymphovascular invasion (Yes vs. No)	283	1.004 (0.628–1.603)	0.987

### High IGF2BP3 expression predicts a poor prognosis in bladder cancer

**Fig. 4:**
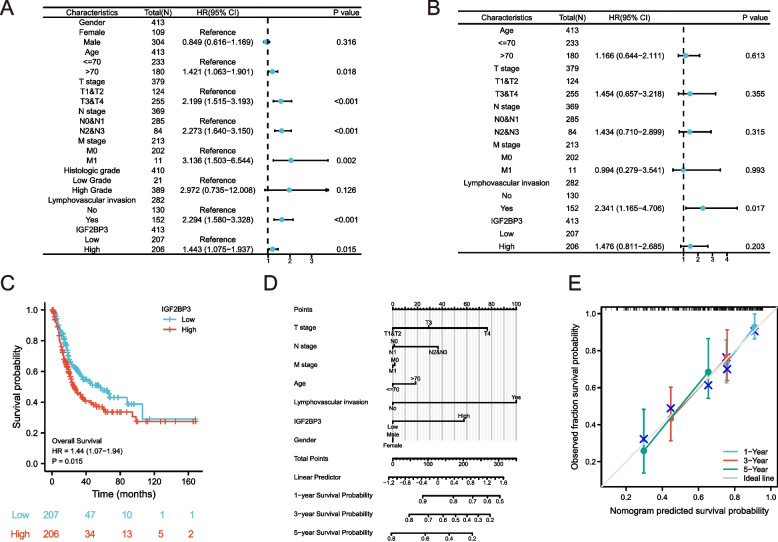
IGF2BP3 serves as an oncogenic role in bladder cancer and high IGF2BP3 expression predicts poor prognosis. **A**-**B** Univariate and multivariate Cox analyses of prognostic factors in bladder cancer. **C** Association between IGF2BP3 expression and OS in bladder cancer patients. **D**, **A** nomogram prognostic model for bladder cancer patients. **E** The nomogram calibration plot indicates that the nomogram was well calibrated

To better understand the potential risk factors associated with the overall survival of patients, we performed univariate and multivariate analyses by the Cox regression model (Fig. 4A-B). The univariate Cox analysis showed that the age (*p* = 0.018), T stage (*p* < 0.001), N stage (*p* < 0.001), M stage (*p* = 0.002), lymphovascular invasion (*p* < 0.001), and IGF2BP3 expression (*p* = 0.015) were all correlated with the overall survival of serous bladder cancer patients; therefore, those factors were included in a multivariate Cox analysis. As shown in Fig. 4B, our results indicated that lymphovascular invasion still had a correlation with the overall survival in the multivariate analysis. Moreover, we investigated the association between mRNA expression of IGF2BP3 and overall survival in bladder cancer patients. Kaplan–Meier survival curves showed that patients with poorer survival had higher levels of IGF2BP3 mRNA expression (HR = 1.44, 95% CI: 1.07–1.94, *P* = 0.015) (Fig. 4C). We constructed a nomogram of OS to integrate IGF2BP3 and other prognostic factors, including age, gender, TNM stage, lymphovascular invasion, and IGF2BP3 expression (Fig. 4D). A higher point on the nomogram represented a worse prognostic factor. The Calibration curve evaluated the nomogram’s performance of IGF2BP3 (Fig. 4E). The nomogram calibration curve demonstrated good agreement between prediction and observation in all cohorts.

### IGF2BP3 related gene enrichment analysis

**Fig. 5:**
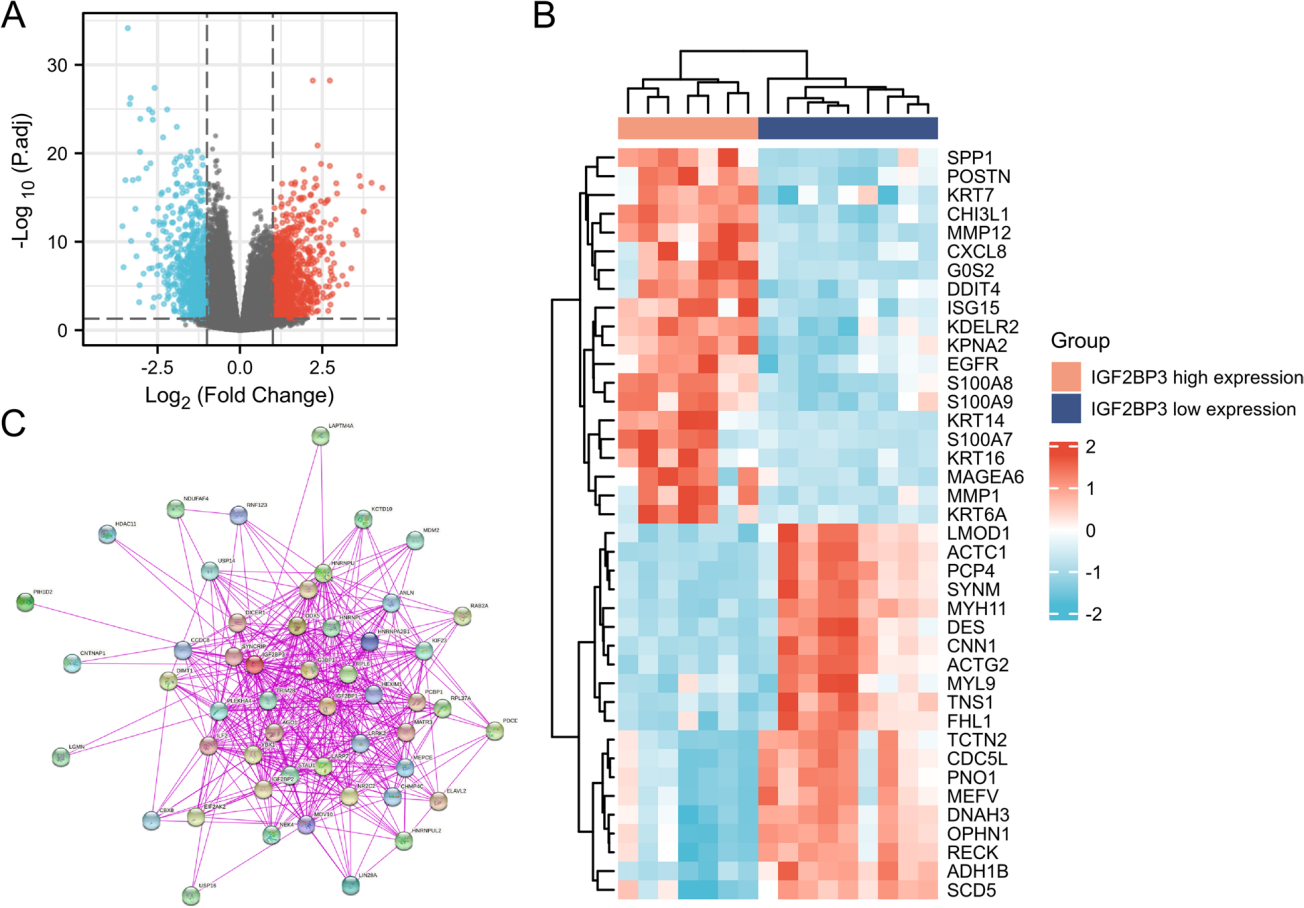
DEGs based on IGF2BP3. **A** Volcano plot visualizing DEGs between high and low expression groups for IGF2BP3. **B** Heatmaps of DEGs based on the expression of IGF2BP3. **C** IGF2BP3 binding proteins obtained by the STRING tool

**Fig. 6:**
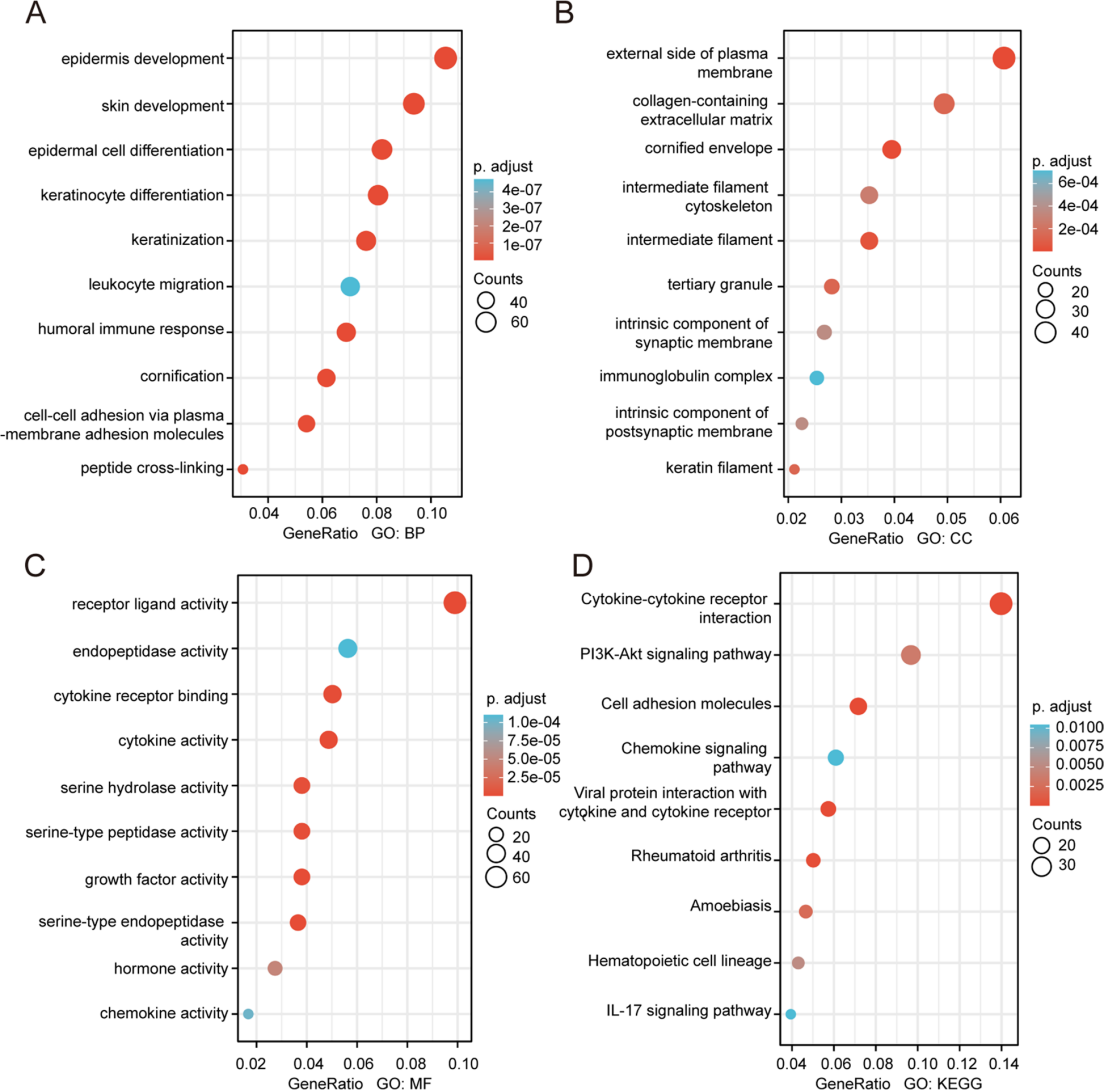
Go and KEGG enrichment analysis of genes related to IGF2BP3 in bladder cancer. **A**-**C** Go enrichment analysis showed the BP (biological processes), CC (cellular components), and MF (molecular function) of co-expressed genes with IGF2BP3. **D** Significantly enriched KEGG terms obtained from KEGG enrichment analysis of co-expressed genes with IGF2BP3

To identify the DEGs based on the expression of IGF2BP3, DEGs were obtained by RNA-seq from the GSE3167 dataset. As shown in Fig. 5A, DEGs were identified. The top 40 DEGs are shown in Fig. 5B. We analyzed the protein–protein interaction networks using data from the STRING database version 11.5 which assesses direct (physical) associations (Fig. 5C). To explore the biological implications of IGF2BP3 related modules, we chose the top 100 most positively correlated genes for enrichment analysis. Gene Ontology–Biological Process (GO–BP) enrichment analysis revealed that the epidermis development and skin development were enriched (Fig. 6A). Gene Ontology–Cellular Component (GO–CC) enrichment analysis revealed that the external side of the plasma membrane and collagen-containing extracellular matrix were enriched (Fig. 6B). Gene Ontology–Molecular Function (GO–MF) enrichment analysis revealed that the receptor-ligand activity and endopeptidase activity were enriched (Fig. 6C). KEGG analysis revealed that cytokine-cytokine receptor interaction was the most significantly enriched pathway (Fig. 6D).

**Fig. 7:**
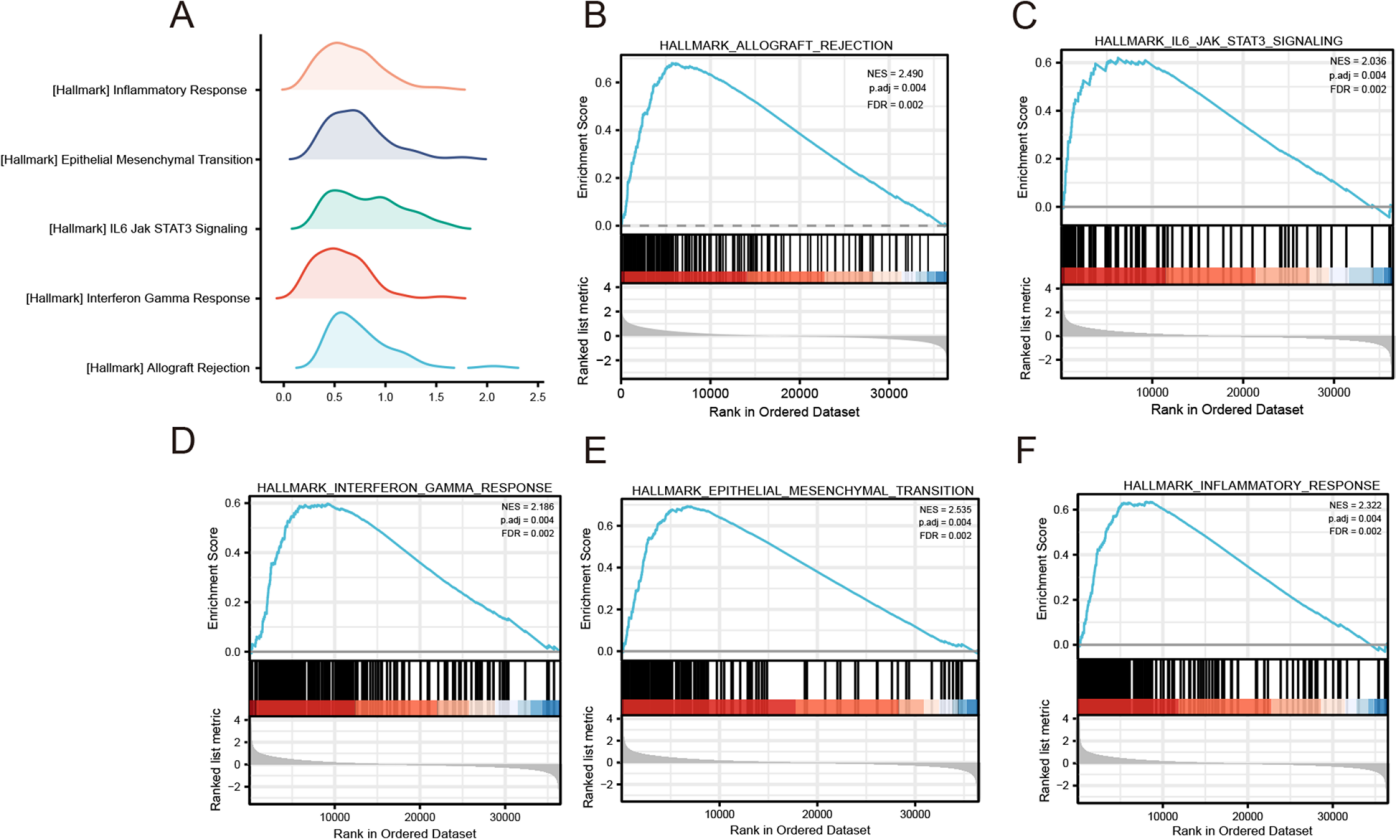
Enrichment plots from the gene set enrichment analysis (GSEA). **A** The ridge plot of GSEA. **B** allograft rejection, **C** interferon-gamma response, **D** IL6 JAK STAT3 signaling, **E** epithelial-mesenchymal transition, **F** inflammatory response were significantly enriched in IGF2BP3-related bladder cancer. NES, normalized enrichment scores; FDR, false discovery rate

We compared the data sets for low and high IGF2BP3 expression using GSEA to identify signaling pathways activated during bladder cancer (Fig. 7A). Multiple pathways, including allograft rejection (Fig. 7B), interferon-gamma response (Fig. 7C), IL6 JAK STAT3 signaling (Fig. 7D), epithelial-mesenchymal transition (EMT) (Fig. 7E), and inflammatory response (Fig. 7F), were significant in IGF2BP3 high-expression phenotype.

### The correlation between IGF2BP3 expression and the infiltration of immune cells

**Fig. 8:**
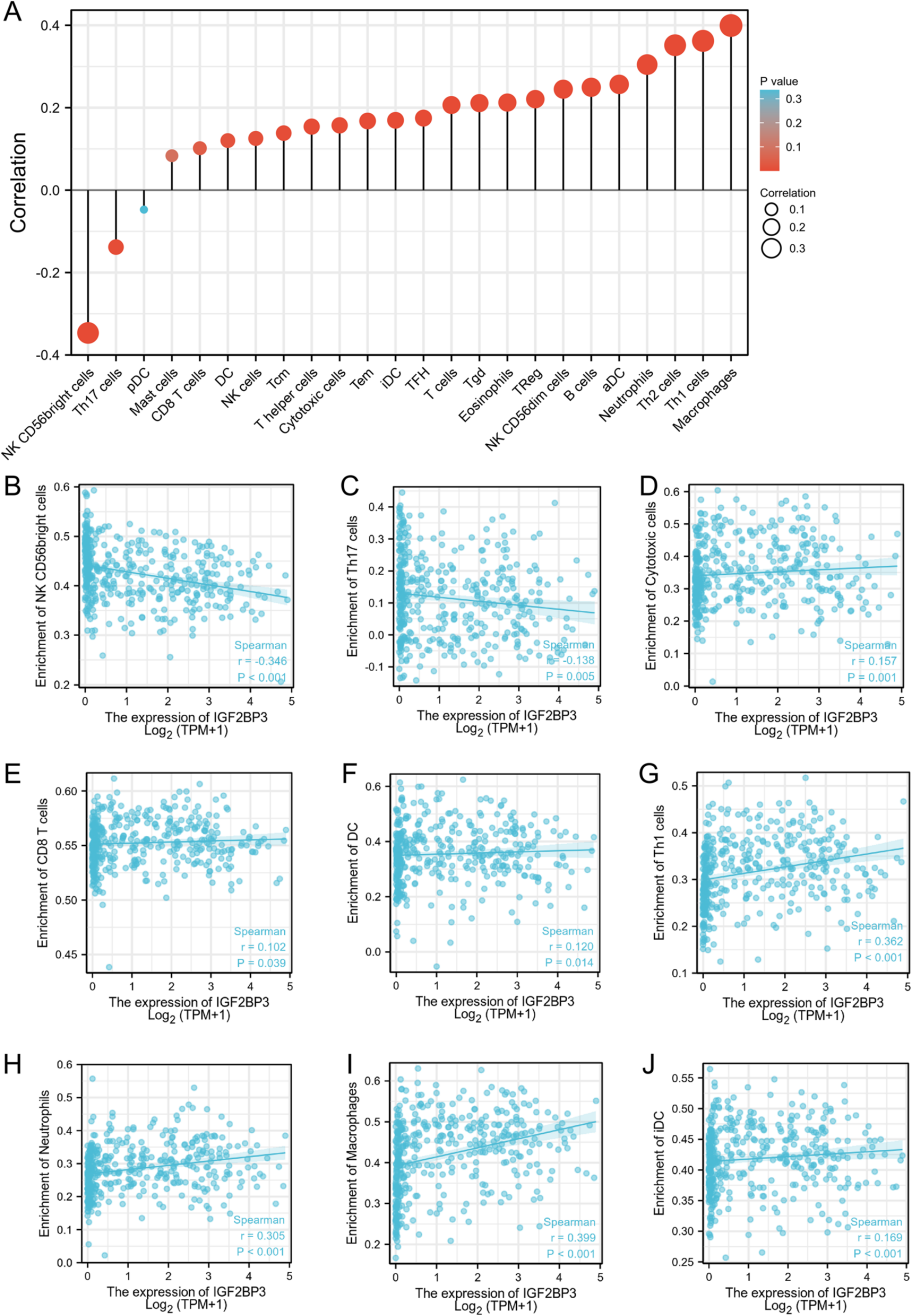
Association analysis of IGF2BP3 gene expression and immune infiltration. **A** The association between IGF2BP3 expression and 24 tumor-infiltrating lymphocytes. **B**-**J** Correlation between IGF2BP3 expression and NK CD56 bright cells, Th17 cells, Cytotoxic cells, CD8 T cells, DC, Th1 cells, Neutrophils, Macrophages, iDC cells

High levels of tumor-infiltrating lymphocytes have been reported to correlate with favorable prognoses in a variety of solid organ malignancies [23, 24]. Therefore, we investigated whether IGF2BP3 expression was correlated with immune infiltration levels in bladder cancer. The correlation between the expression level of IGF2BP3 and immune cell infiltration level quantified by ssGSEA was analyzed by Spearman correlation (Fig. 8A). In addition, we found that the expression of IGF2BP3 is negatively correlated with infiltration levels of NK CD56dim cells (*R* = -0.346, *p* < 0.001, Fig. 8B) and Th17 cells (*R* = -0.138, *p* = 0.005, Fig. 8C). Moreover, IGF2BP3 expression was positively correlated with infiltration level of Cytotoxic cells, CD8 T cells, DC, Th1 cells, Neutrophils, Macrophages, iDC cells (Fig. 8D-J).

**Fig. 9:**
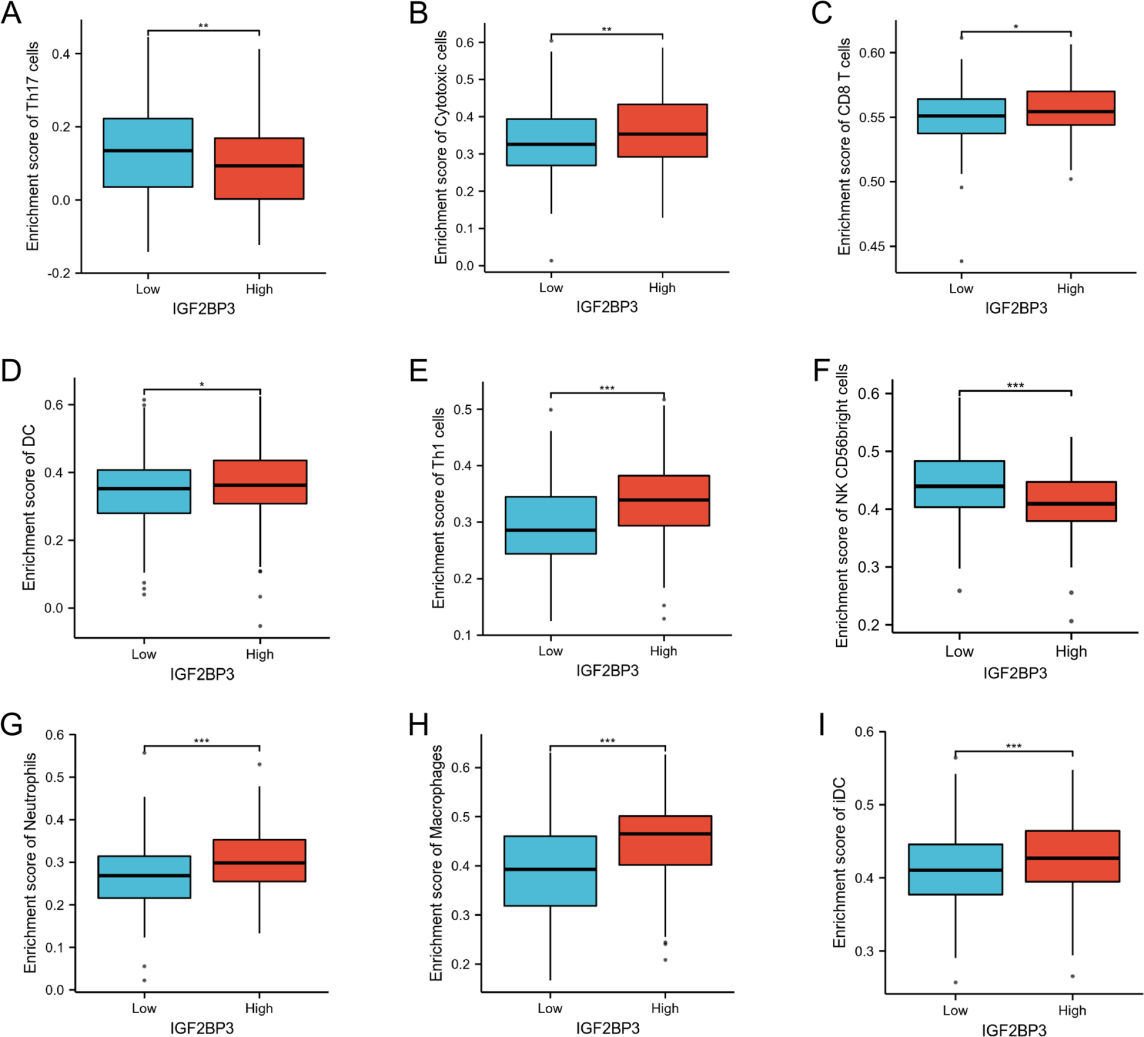
Comparison of immune cells between high– and low–IGF2BP3 expression groups. **A**-**I** Histogram showing the difference of Th17 cells, Cytotoxic cells, CD8 T cells, DC, Th1 cells, NK CD56 bright cells, Neutrophils, Macrophages, and iDC cells infiltration level between high–and low–IGF2BP3 expression groups

**Fig. 10:**
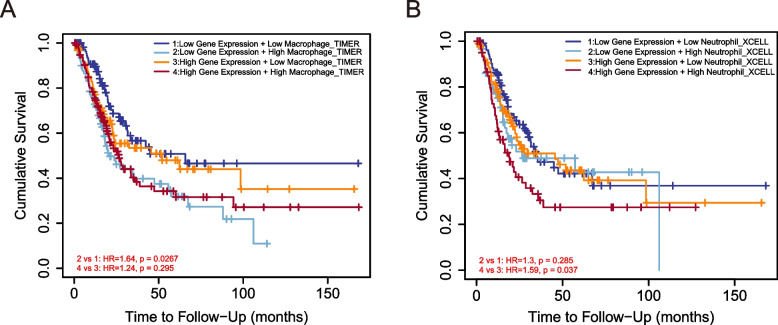
Impact of immune cell infiltration on prognosis in bladder cancer patients. **A** Clinical survival outcome of bladder cancer patients in the high macrophage group. **B** Clinical survival outcome of bladder cancer patients in the high–Neutrophil group

These data prompted us to investigate the potential relationship between IGF2BP3 expression level and immune infiltration. We found that when patients were categorized into high, and low expression of IGF2BP3, there was a strong significant correlation between IGF2BP3 expression levels and Th17 cells, cytotoxic cells, CD8 T cells, DC, Th1 cells, NK CD56bright cells, Neutrophils, Macrophages and iDC cells (Fig. 9A-I). Next, we used TIMER analysis to identify the association of tumor‐infiltrating immune cells with survival outcomes of bladder cancer patients. The results indicated that high levels of macrophage and neutrophil infiltration have been associated with poor prognosis of bladder cancer (Fig. 10A-B).

### High expression of IGF2BP3 increased PD-L1 expression in bladder cancer

**Fig. 11:**
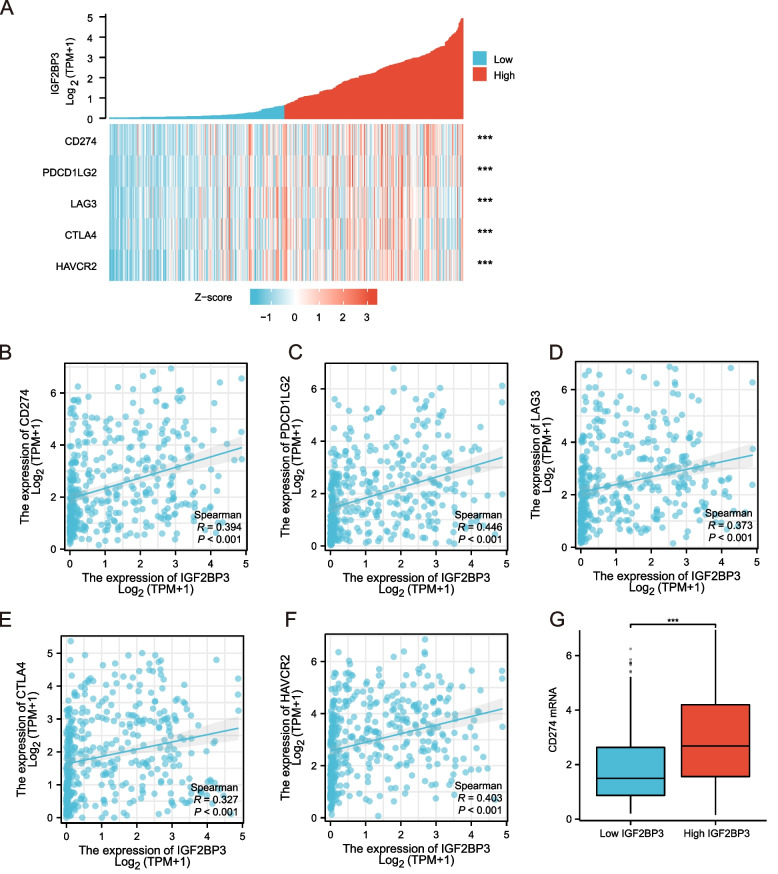
IGF2BP3 expression was significantly associated with PD-L1 expression. **A** heatmap of immune checkpoints based on IGF2BP3 expression. **B**-**F** the correlation between IGF2BP3 expression and CD274 (PDL-1), PDCD1LG2 (PDL-2), LAG3, CTLA4, and HAVCR2. G, PD-L1 mRNA expression increased by IGF2BP3 upregulation

We assumed that IGF2BP3 correlates with the overexpression of the immune checkpoint molecule PD-L1, potentially contributing to the development of immune exhaustion. Heatmap and correlations between IGF2BP3 and immune checkpoints including PD-L1, PD-L2, LAG3, CTLA4, and TIM3 were shown in Fig. 11A. We found significant positive correlations between the IGF2BP3 expression and PD-L1, PD-L2, LAG3, CTLA4, and TIM3 expression in bladder cancer (Fig. 11B-F). Moreover, elevated level of IGF2BP3 mRNAs is correlated with a concomitant increase of PD-L1 mRNA (Fig. 11G). Furthermore, RT–qPCR analyses showed that endogenous CD274 (PDL-1), PDCD1LG2 (PDL-2), LAG3, CTLA4, and HAVCR2 mRNA levels in 5637 cells were increased upon IGF2BP3 overexpression compared with the control cells. On contrary, IGF2BP3 knockdown in T24 cells substantially decreased CD274 (PDL-1), PDCD1LG2 (PDL-2), LAG3, CTLA4, and HAVCR2 mRNA expression (Figure S1).

### Verification of IGF2BP3 up-expression in bladder cancer tissues compared to normal tissues

**Fig. 12:**
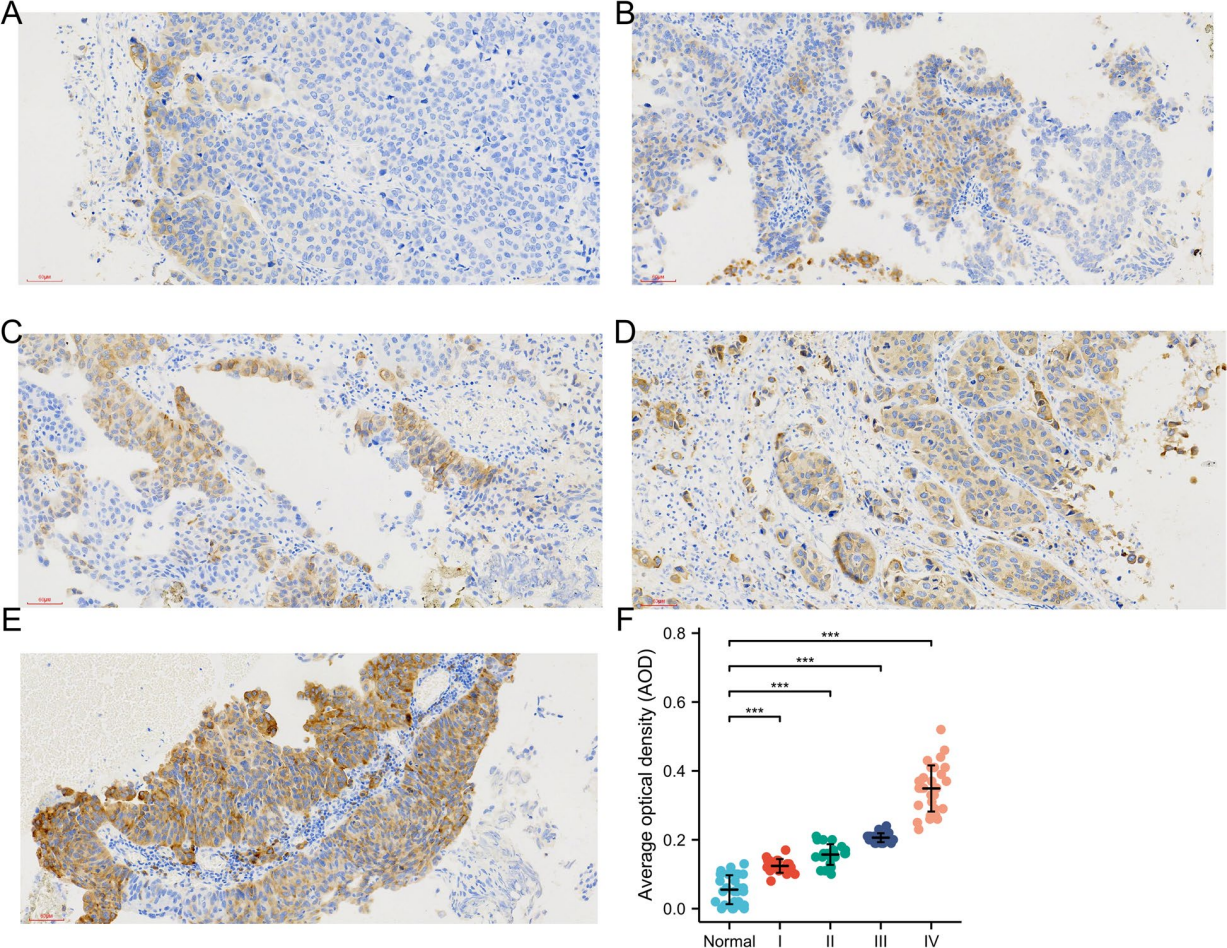
Expression of IGF2BP3 in different clinical stage of bladder cancer tissues and normal bladder tissues. **A**-**E** Expression of IGF2BP3 in paraffin-embedded tissue sections of normal bladder tissues (*n* = 27), stage I bladder cancer tissues (*n* = 20), stage II bladder cancer tissues (*n* = 22), stage III bladder cancer tissues (*n* = 22), and stage IV bladder cancer tissues (*n* = 31) was analyzed with immunohistochemistry. **F** Quantification of the average optical density (AOD) for IGF2BP3 in normal bladder tissue and different clinical stages of bladder cancer

We performed immunohistochemical staining for IGF2BP3 expression of formalin-fixed paraffin embedded primary bladder cancer of 95 patients who underwent resection at our institution. Twenty of the tumors were Stage I, twenty-two were Stage II, twenty-two were Stage III and thirty-one were Stage IV. Twenty-seven bladder normal mucosa samples were used as controls. As shown in Figs. 12A-E, positive IGF2BP3 staining was mainly detected in the cytoplasmic in the bladder cancer tissues. The protein level of IGF2BP3 was significantly elevated in bladder carcinoma samples compared with normal tissue. In addition, our results showed a positive correlation between the expression level of IGF2BP3 and the bladder cancer stage (Fig. 12F). The overall survival rate of bladder cancer patients with higher IGF2BP3 expression in tumors was significantly poorer than that of patients with lower IGF2BP3 expression in tumors (*P* = 0.024) (Figure S2). We further evaluated CD274 (PDL-1), CD68, CD16, and CD3 expression in the *n* = 95 clinical cohort using immunohistochemistry. As shown in Figure S3, CD274 (PDL-1), CD68, CD16, and CD3 expression were observed in the bladder cancer tissues. Quantification of the average optical density (AOD) of CD274 (PDL-1), CD68, CD16, and CD3 is higher in the high expression of IGF2BP3 patients than that in the low expression of IGF2BP3 patients (Figure S4).

### IGF2BP3 promotes the migration and invasion of bladder cancer cells

**Fig. 13:**
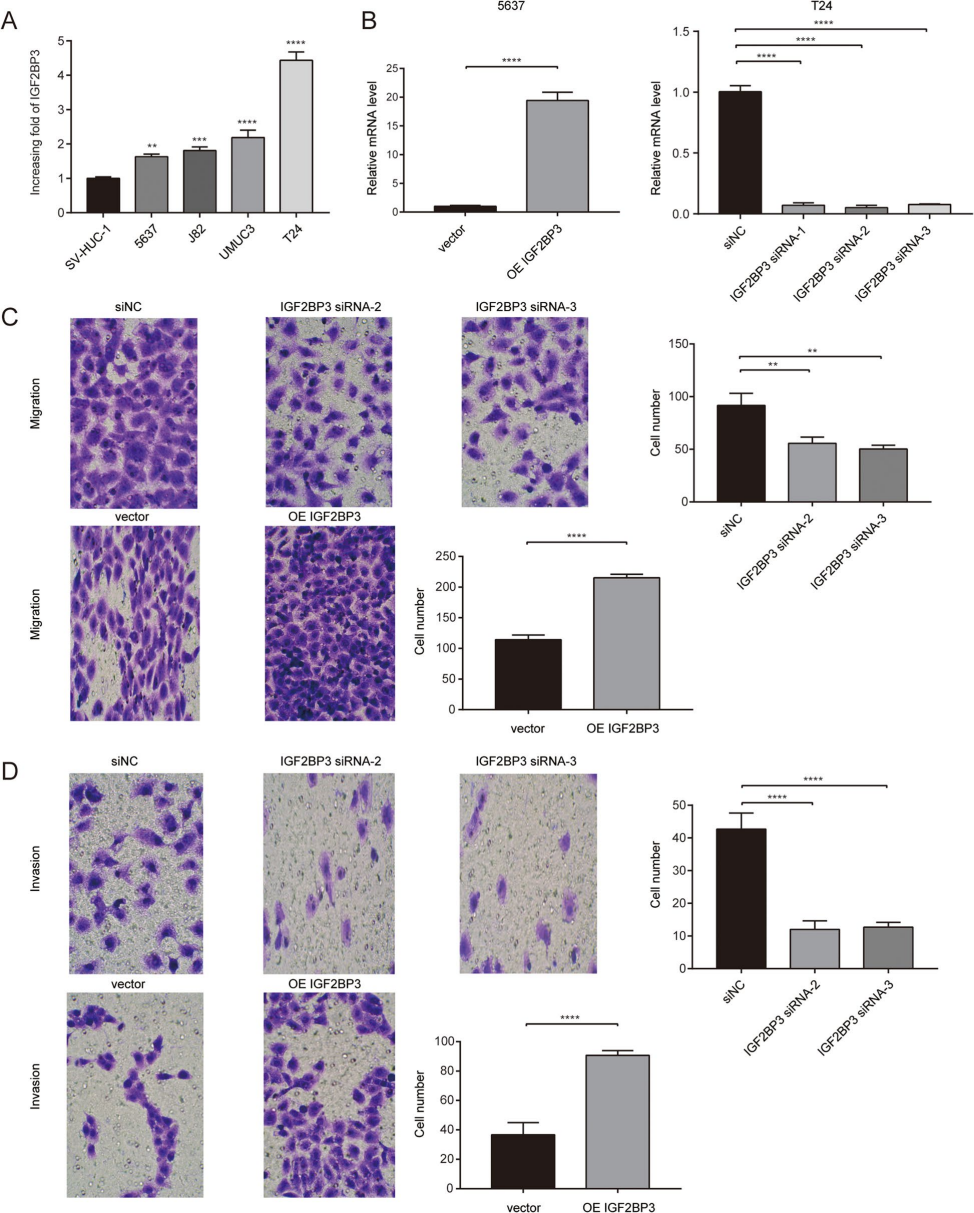
IGF2BP3 promotes migration and invasion of bladder cancer cells. **A** Real-time PCR analysis of IGF2BP3 expression in SV-HUC-1 and bladder cancer cell lines. **B** Real-time PCR analysis of indicated bladder cancer cells transfected with IGF2BP3-vector, IGF2BP3, IGF2BP3-siRNA-vector, IGF2BP3-siRNA-1, IGF2BP3-siRNA-2 or IGF2BP3-siRNA-3. **C**-**D** IGF2BP3 knockdown inhibits migration and invasion in T24 cells, while IGF2BP3 overexpression promotes migration and invasion in 5637 cells

To further explore the role of IGF2BP3 in bladder cancer cell metastasis, transwell migration and invasion assays were performed. The expression of IGF2BP3 in normal human bladder epithelial cells (SV-HUC-1) and bladder cancer cell lines (5637, J82, UMUC3 and T24) was validated by qRT-PCR. Bladder cancer cell lines showed a relatively high level of IGF2BP3 compared with SV-HUC-1 cells (Fig. 13A). Next, we down-regulated and up-regulated IGF2BP3 expression using siRNA and lentivirus transfection assay, respectively. IGF2BP3 mRNA expression level was significantly downregulated and upregulated compared with the control group in T24 and 5637 cells, respectively (Fig. 13B). As shown in Fig. 13C and D, silencing IGF2BP3 expression decreased the number of migrated and invaded bladder cancer cells while ectopic expression of IGF2BP3 significantly enhanced the migrated and invaded rate of bladder cancer cells. Thus, these data suggest that IGF2BP3 promotes the migratory and invasive abilities of bladder cancer cells.

## Discussion

Bladder cancer is a malignant disease with a high incidence and mortality rate [25]. Therefore, identifying novel biomarkers and further understanding the molecular mechanisms of bladder cancer are crucial for improved diagnostic strategies and treatment options. According to previous studies, IGF2BP3 can be a valuable prognostic marker in human cancer [26–28]. In this present study, we attempted to investigate the biological function and molecular mechanism of IGF2BP3 and assess its feasibility as potential cancer biomarker.

Analysis of a pan-cancer project showed that IGF2BP3 was amplified in most human cancer types. Furthermore, increased expression of IGF2BP3 significantly correlated with reduced overall survival for patients with bladder cancer. Our Cox regression analysis demonstrated that IGF2BP3 was independently prognostic for OS in bladder cancer. IGF2BP3 ought to be a promising diagnostic biomarker based on the above results and ROC analysis.

The tumor microenvironment consists of different types of immune cells such as T cells, dendritic cells and NK cells that kill cancer cells [29]. The microenvironment is known to play an important role in tumor initiation, progression, and metastasis [30, 31]. During cancer development, the tumor microenvironment with infiltrating immune and non-immune cells, as well as the extracellular matrix undergoes substantial changes that can influence tumor progression [32, 33]. Gene enrichment analysis indicated that IGF2BP3 was mainly involved in immune and inflammatory responses. Our study found that IGF2BP3 expression in bladder cancer is correlated with the type and density of tumor-infiltrating immune cells. We further assumed that IGF2BP3 may educate inflammatory tumor microenvironment to promote tumor progression by orchestrating crosstalk between tumor cells and their microenvironment. Pan et al. reported that circNEIL3 can promote tumor-associated macrophages to acquire immunosuppressive properties by stabilizing IGF2BP3 in glioma [34]. We also demonstrate a significant positive association between the extent of macrophage infiltration and the expression of IGF2BP3. Tumor-associated macrophages, typically with characteristics of activated macrophages, are an important component of the tumor microenvironment and correlate with poor prognosis in other tumor types [35]. Remarkably, we found that high infiltration of macrophages and neutrophils was associated with poor prognosis. Certainly, other cell types in the tumor microenvironment also affect tumor cell invadopodium formation and intravasation, revealing the complexity of the tumor microenvironment. The relationship between IGF2BP3 expression and immunoregulatory cells requires further study.

The high mutational burden of bladder cancer makes it susceptible to immunotherapy, particularly with checkpoint inhibitors, monoclonal antibodies against programmed cell death-1 (PD-1) and its ligand, PD-L1 [36, 37]. It is well-known that immune checkpoint signaling is an important mechanism of immune evasion in tumors [38]. Our results indicated a positive correlation between IGF2BP3 expression and the expression of immune checkpoints, such as PD-L1, PD-L2, LAG3, CTLA4, and TIM3. Along with the decreasedIGF2BP3 mRNA levels, there was also a significant down-regulation of PD-L1 mRNA expression, indicating that IGF2BP3 may participate in the immune response of bladder cancer through PD-L1. Wan et al. showed that METTL3/m6A/IGF2BP3 signaling axis mediates PD-L1 mRNA activation and further inhibits tumor immune surveillance [39].

GSEA data analysis revealed that the EMT pathway was significantly enriched in the IGF2BP3 high expression group. In bladder cancer, immunohistochemistry staining analysis showed that there is a statistically significant correlation exists between IGF2BP3 expression and tumor staging. These results indicate that IGF2BP3 is involved in bladder cancer progression. Xu et al. reported that IGF2BP3 knockdown inhibited the migration ability of colorectal cancer cells via inducing epithelial-mesenchymal transition [7]. Consistent with these results, we also observed that IGF2BP3 knockdown significantly inhibited the ability of migration and invasion in bladder cancer cell line, whileits overexpression has the opposite effect.

Our findings demonstrate that IGF2BP3 is overexpressed in bladder cancer and correlated with poor survival outcomes. Both IGF2BP3 and immune cell infiltration play critical roles in the development and progression of bladder cancer. In the present study, we identified IGF2BP3 as a prognostic marker and promising therapeutic target in bladder cancer.

There are some limitations in our study. First, it remains to be determined whether IGF2BP3 regulates EMT and immune response directly or indirectly. Second, the results of the migration and invasion assays in vitro experiments still need to be verified in animal models. Third, this study provides a foundation for further studies of the correlation between IGF2BP3 and the tumor-associated immune microenvironment. However, additional studies are needed to further validate this hypothesis.

## Conclusions

IGF2BP3 overexpression was related to disease progression and poor prognosis, as well as infiltration of immune cells in bladder cancer. IGF2BP3 can be a promising independent prognostic biomarker and potential treatment target for bladder cancer.

## Supplementary Information

**Additional file 1:** **Table S1.** Clinicopathologic characteristics of 95patients with bladder cancer. **Figure S1.** Regulation of CD274 (PDL-1), PDCD1LG2(PDL-2), LAG3, CTLA4, and HAVCR2 by IGF2BP3. A-B, Expression of IGF2BP3 in T24and 5637 cells that were transfected with indicated vectors was determined byRT–qPCR. IGF2BP3 overexpression increased CD274 (PDL-1) (Figure C), PDCD1LG2 (PDL-2)(Figure D), LAG3 (Figure E), CTLA4 (Figure F), and HAVCR2 (Figure G) mRNA levelin 5637 cells. IGF2BP3 silencing decreased CD274 (PDL-1) (Figure H), PDCD1LG2(PDL-2) (Figure I), LAG3 (Figure J), CTLA4 (Figure K), and HAVCR2 (Figure L)mRNA level in T24 cells. **Figure S2.** Kaplan-Meier overall survival curves forall 95 patients with bladder cancer stratified by high and low expression of IGF2BP3. **Figure S3.** Representative images of CD274 (PDL-1)(Figure A), CD68 (Figure B), CD16 (Figure C), and CD3 (Figure D) in bladdercancer tissues by immunohistochemistry. **Figure S4.** IGF2BP3 expression was significantlyassociated with CD274, CD68, CD16, and CD3 expression by immunohistochemistry.Quantification of the average optical density (AOD) of CD274 (PDL-1) (Figure A),CD68 (Figure B), CD16 (Figure C), and CD3 (Figure D) is higher in the highexpression of IGF2BP3 patients than that in the low expression of IGF2BP3patients.

## Data Availability

The datasets of this study are available on request to the corresponding author.

## Abbreviations

ACC: Adrenocortical carcinoma
BLCA: Bladder Urothelial Carcinoma
BRCA: Breast invasive carcinoma
CESC: Cervical and endocervical cancer
CHOL: Cholangiocarcinoma
COAD: Colon adenocarcinoma
DLBC: Lymphoid Neoplasm Diffuse Large B-cell Lymphoma
ESCA: Esophageal carcinoma
GBM: Glioblastoma multiforme
HNSC: Head and neck cancer
KICH: Kidney chromophobe
KIRC: Kidney renal clear cell carcinoma
KIRP: Kidney renal papillary cell carcinoma
LAML: Acute Myeloid Leukemia
LGG: Brain Lower Grade Glioma
LIHC: Liver hepatocellular carcinoma
LUAD: Lung adenocarcinoma
LUSC: Lung squamous cell carcinoma
MESO: Mesothelioma
OV: Ovarian serous cystadenocarcinoma
PAAD: Pancreatic adenocarcinoma
PCPG: Pheochromocytoma and paraganglima
PRAD: Prostate adenocarcinoma
READ: Rectum adenocarcinoma
SARC: Sarcoma
SKCM: Skin Cutaneous Melanoma
STAD: Stomach adenocarcinoma
TGCT: Testicular Germ Cell Tumors
THCA: Thyroid carcinoma
THYM: Thymoma
UCEC: Uterine corpus
UCS: Uterine Carcinosarcoma
UVM: Uveal Melanoma
TIMER: Tumor Immune Estimate Resource
TCGA: The Cancer Genome Atlas
GEO: Gene Expression Omnibus
IHC: Immunohistochemistry
ROC: Receiver operating characteristic
AUC: Area under curve
OS: Overall survival
DFS: Disease free survival
DSS: Disease specific survival
PFI: Progress free interval
GO: Gene ontology
BP: Biological process
CC: Cellular component
MF: Molecular function
GSEA: Gene Set Enrichment Analysis
NES: Normalized enrichment score
FDR: False discovery rate
