# Supplementary material for: IGF2BP3 overexpression predicts poor prognosis and correlates with immune infiltration in bladder cancer
Source: BMC Cancer. 2023 Feb 3;23:116. doi: 10.1186/s12885-022-10353-5 (PMC9896754; doi:10.1186/s12885-022-10353-5)

Table S1 Clinicopathologic characteristics of 95 patients with bladder cancer

| Characteristic | Number of cases |
| --- | --- |
| Gender, n (%) |  |
| Female | 34 (35.8%) |
| Male | 61 (64.2%) |
| Age, n (%) |  |
| <=70 | 35 (36.8%) |
| >70 | 60 (63.2%) |
| T stage, n (%) |  |
| T1 | 20 (21.0%) |
| T2 | 40 (42.1%) |
| T3 | 26 (27.4%) |
| T4 | 9 (9.5%) |
| N stage, n (%) |  |
| N0 | 73 (76.8%) |
| N1 | 6 (6.4%) |
| N2 | 15 (15.8%) |
| N3 | 1 (1.0%) |
| M stage, n (%) |  |
| M0 | 90 (94.7%) |
| M1 | 5 (5.3%) |
| Histologic grade, n (%) |  |
| High Grade | 67 (70.5%) |
| Low Grade | 28 (29.5%) |
| Lymphovascular invasion, n (%) |  |
| No | 73 (76.8%) |
| Yes | 22 (23.2%) |
| AJCC stage, n (%) |  |
| Stage I | 20 (21.0%) |
| Stage II | 22 (23.2%) |
| Stage III | 22 (23.2%) |
| Stage IV | 31 (32.6%) |
| Expression of IGF2BP3, n (%) |  |
| Low expression | 31 (32.6%) |
| High expression | 64 (67.4%) |

Figure S1: Regulation of CD274 (PDL-1), PDCD1LG2 (PDL-2), LAG3, CTLA4, and HAVCR2 by IGF2BP3. A-B, Expression of IGF2BP3 in T24 and 5637 cells that were transfected with indicated vectors was determined by RT–qPCR. IGF2BP3 overexpression increased CD274 (PDL-1) (Figure C), PDCD1LG2 (PDL-2) (Figure D), LAG3 (Figure E), CTLA4 (Figure F), and HAVCR2 (Figure G) mRNA level in 5637 cells. IGF2BP3 silencing decreased CD274 (PDL-1) (Figure H), PDCD1LG2 (PDL-2) (Figure I), LAG3 (Figure J), CTLA4 (Figure K), and HAVCR2 (Figure L) mRNA level in T24 cells.


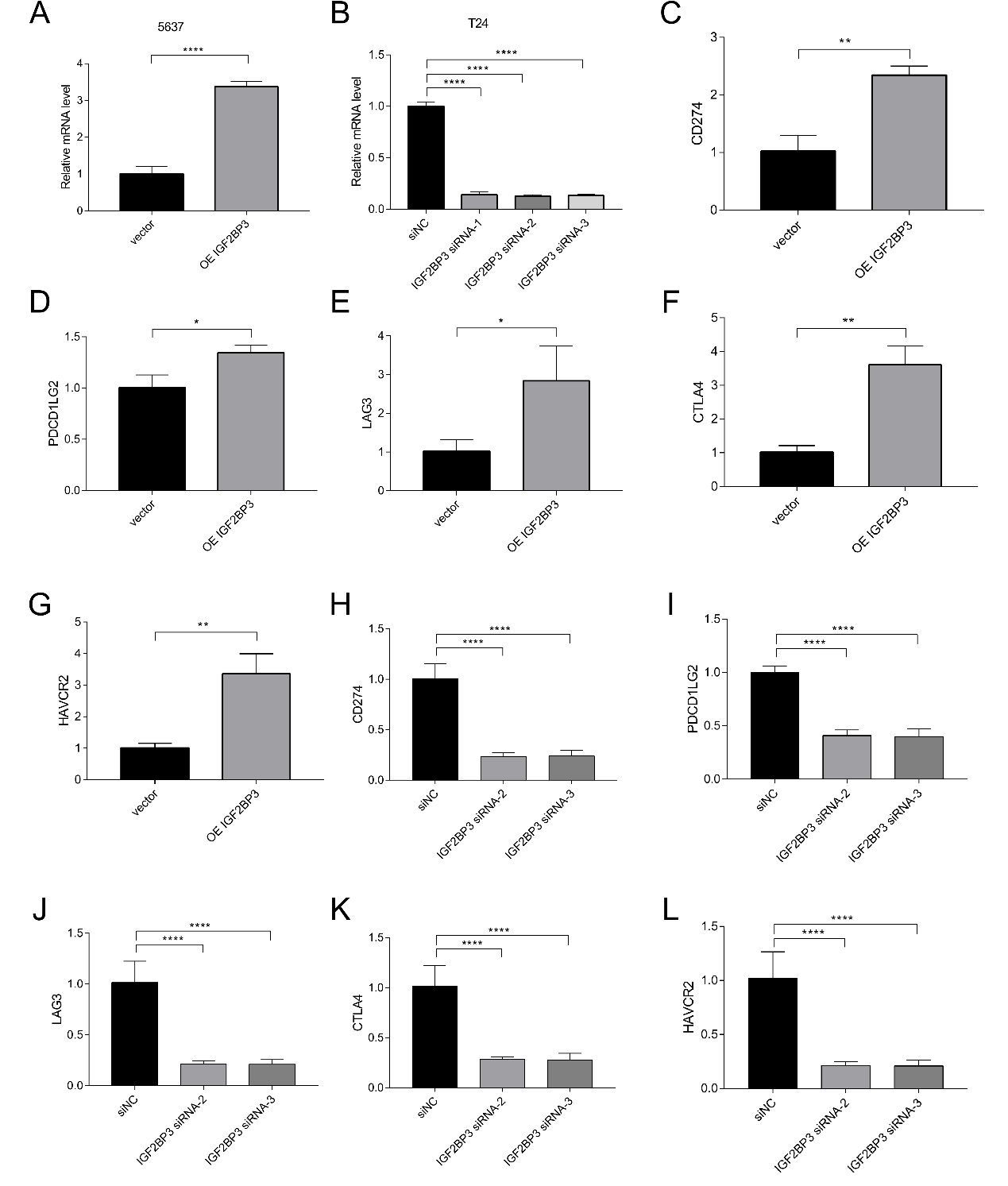


Figure S2: Kaplan-Meier overall survival curves for all 95 patients with bladder cancer stratified by high and low expression of IGF2BP3.


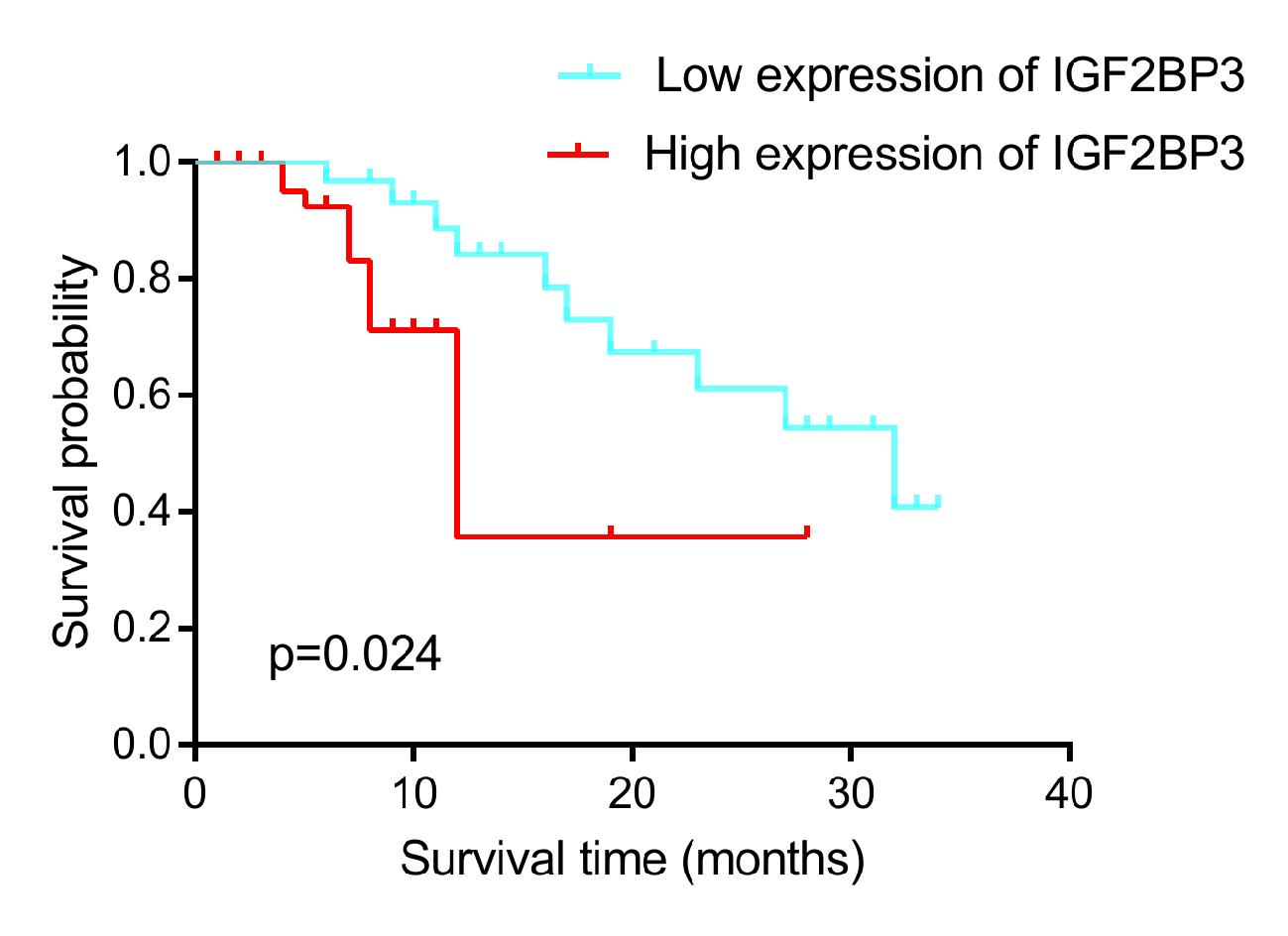


Figure S3: Representative images of CD274 (PDL-1) (Figure A), CD68 (Figure B), CD16 (Figure C), and CD3 (Figure D) in bladder cancer tissues by immunohistochemistry.


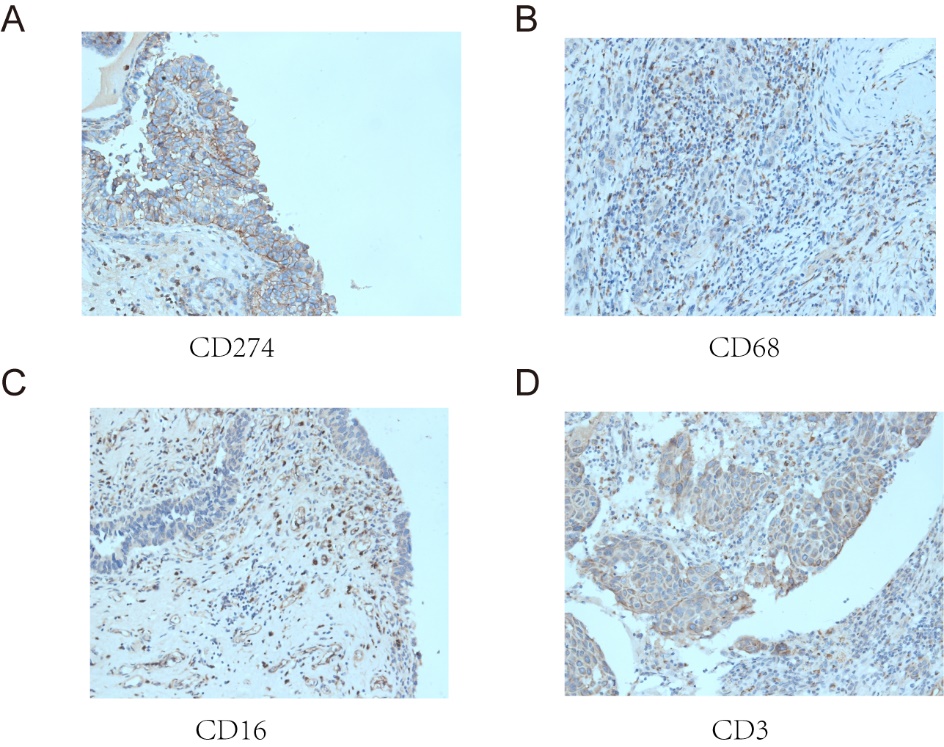


Figure S4: IGF2BP3 expression was significantly associated with CD274, CD68, CD16, and CD3 expression by immunohistochemistry. Quantification of the average optical density (AOD) of CD274 (PDL-1) (Figure A), CD68 (Figure B), CD16 (Figure C), and CD3 (Figure D) is higher in the high expression of IGF2BP3 patients than that in the low expression of IGF2BP3 patients.


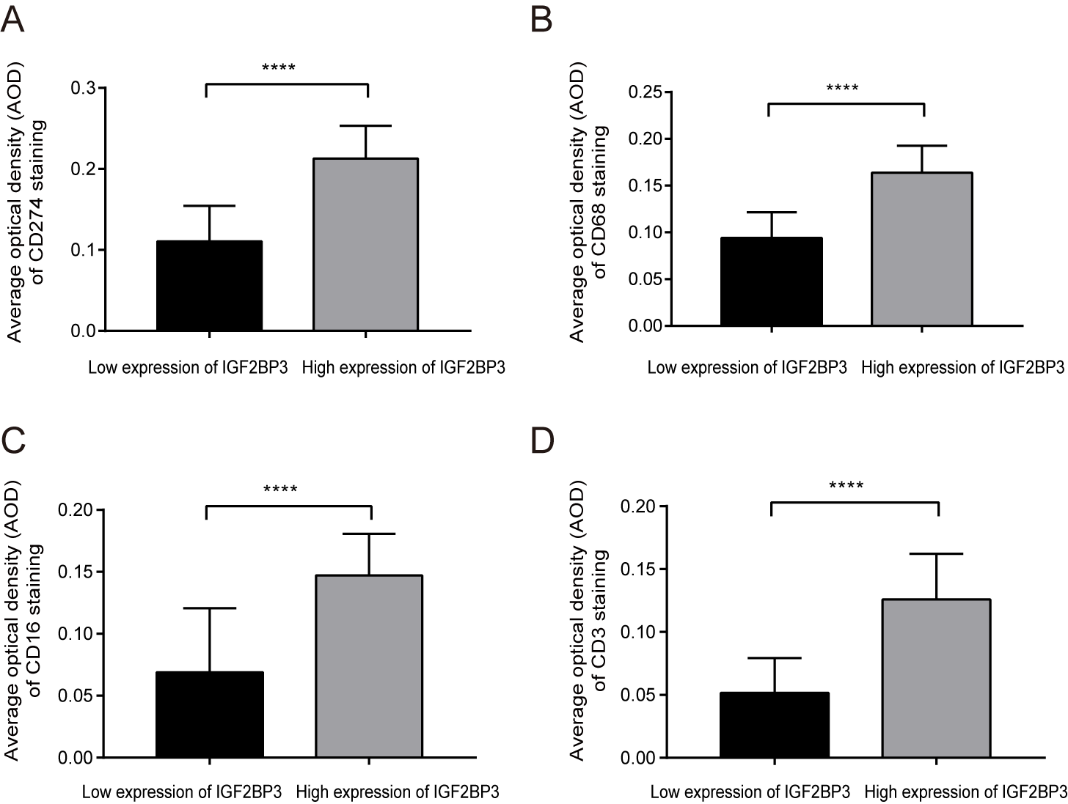

Supplement: Supplementary file 1 — Additional file 1: Table S1. Clinicopathologic characteristics of 95patients with bladder cancer. Figure S1. Regulation of CD274 (PDL-1), PDCD1LG2(PDL-2), LAG3, CTLA4, and HAVCR2 by IGF2BP3. A-B, Expression of IGF2BP3 in T24and 5637 cells that were transfected with indicated vectors was determined byRT–qPCR. IGF2BP3 overexpression increased CD274 (PDL-1) (Figure C), PDCD1LG2 (PDL-2)(Figure D), LAG3 (Figure E), CTLA4 (Figure F), and HAVCR2 (Figure G) mRNA levelin 5637 cells. IGF2BP3 silencing decreased CD274 (PDL-1) (Figure H), PDCD1LG2(PDL-2) (Figure I), LAG3 (Figure J), CTLA4 (Figure K), and HAVCR2 (Figure L)mRNA level in T24 cells. Figure S2. Kaplan-Meier overall survival curves forall 95 patients with bladder cancer stratified by high and low expression of IGF2BP3. Figure S3. Representative images of CD274 (PDL-1)(Figure A), CD68 (Figure B), CD16 (Figure C), and CD3 (Figure D) in bladdercancer tissues by immunohistochemistry. Figure S4. IGF2BP3 expression was significantlyassociated with CD274, CD68, CD16, and CD3 expression by immunohistochemistry.Quantification of the average optical density (AOD) of CD274 (PDL-1) (Figure A),CD68 (Figure B), CD16 (Figure C), and CD3 (Figure D) is higher in the highexpression of IGF2BP3 patients than that in the low expression of IGF2BP3patients. [file 12885_2022_10353_MOESM1_ESM.docx]
